# Bioinspired Janus Membrane with Dopamine-ZnO Coating for Antibacterial Filtration in Oral Applications

**DOI:** 10.3390/polym17101356

**Published:** 2025-05-15

**Authors:** Yumeng Guo, Qian Wang, Guoming Sun, Ying Zheng

**Affiliations:** 1 School of Stomatology, Capital Medical University, Beijing 100070, China; ymguo@mail.ccmu.edu.cn (Y.G.);; 2 Department of Stomatology, Peking Union Medical College Hospital, Peking Union Medical College, Chinese Academy of Medical Sciences, Beijing 100730, China; 3 Zhejiang Sci-Tech University Shengzhou Innovation Research Institute, Shengzhou 312400, China

**Keywords:** zinc oxide nanoparticle, barrier membrane, antibacterial, Janus membrane, filtration, dental biomaterial

## Abstract

Developing an oral fibrous barrier membrane that prevents bacterial invasion while possessing antibacterial properties and facilitating fluid decompression remains a significant clinical and scientific challenge. In this study, we developed a novel Janus membrane by modifying a polypropylene (PP) fibrous membrane with dopamine and zinc oxide nanoparticles (ZnO-NPs). Fabricated via a simple floating immersion method, this asymmetric bilayer structure consists of a hydrophobic PP layer and a hydrophilic PP/dopamine@30 nm ZnO layer, providing both antibacterial properties and enhanced fluid filtration. The mechanical properties of the PP/ZnO membrane were significantly enhanced, with an increase in the Young’s modulus and ultimate tensile strength, indicating improved strength. Antibacterial activity against *Streptococcus mutans* (*S. mutans*) demonstrated a significant reduction in biofilm formation on the PP/dopamine@30 nm ZnO surface compared to unmodified PP. Water flux tests confirmed a stable, high filtration rate, with increased permeability under rising pressure. In vivo experiments with miniature pigs confirmed reduced bacterial presence on the sterile side of the membrane. These findings highlight the potential of the membrane for oral exudate filtration, extending filtration time and minimizing infection risks under strict sterility conditions. Further improvements in barrier properties are necessary to optimize its clinical performance.

## 1. Introduction

Fibrous membranes are widely utilized in tissue engineering due to their ability to manage fluid dynamics effectively [1,2]. In skin tissue engineering, for instance, excessive exudate accumulation can exacerbate inflammation and delay wound healing [3]. To mitigate these effects, fibrous membranes are commonly employed in wound care to absorb inflammatory fluid, alleviate inflammation, and promote tissue repair and regeneration [4,5]. However, their application in the oral environment presents significant challenges. The oral cavity harbors the second most diverse microbial community in the body, consisting of more than 700 microbial species [6]. Additionally, its warm, moist and nutrient-rich conditions create an ideal environment for bacterial proliferation, complicating the management of infections [7].

In oral clinical treatment, particularly in pathophysiological conditions such as irreversible pulpitis, inflammatory fluid accumulates within the pulp chamber [8], leading to increased pressure and eventual pulp necrosis if left unmanaged [9]. Therefore, effective decompression of inflammatory fluid in the early stages is crucial for preventing further complications [10,11,12]. Current barrier membranes, including expanded polytetrafluoroethylene (e-PTFE), dense polytetrafluoroethylene (d-PTFE), and collagen-based membranes such as the Geistlich Bio-Gide and Jason membranes, which are primarily designed for tissue repair rather than fluid management, lack antibacterial properties [13,14]. Furthermore, studies on the use of barrier membranes for fluid control in dentistry remain limited. As a result, developing an oral fibrous barrier membrane that not only prevents bacterial invasion but also possesses antibacterial properties and facilitates fluid decompression presents a significant clinical and scientific challenge.

Based on these ideal properties, we were drawn to the concept of developing a dual-functional membrane. The idea was to create a membrane that could serve two distinct purposes: preventing external contamination and facilitating the release of exudate. One promising candidate that aligns with this concept is the Janus membrane, characterized by its asymmetric structure, with one hydrophobic and one hydrophilic side [15,16]. This unique design has garnered attention in biomedical applications, including hemostasis, wound healing, guided bone regeneration, and artificial peritoneums [1,17,18,19,20]. The hydrophobic side prevents external contamination, while the hydrophilic side absorbs and facilitates the release of exudate. For example, Zhang et al. developed a photoresponsive Janus nanofiber dressing via electrospinning, constructing an asymmetric membrane with polyacrylonitrile fibers doped with polydopamine (PDA) on a PP nonwoven substrate [21]. This design enabled rapid exudate removal within 22 s, thus accelerated wound healing in diabetic conditions.

**Scheme 1:**
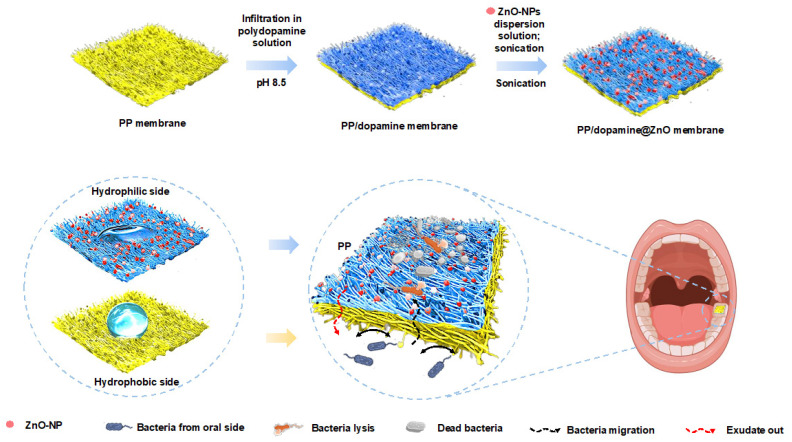
Schematic diagram of the PP/dopamine@30 nm ZnO Janus membrane. The PP fibrous membrane was floated on a dopamine solution for 24 h, then immersed in a 30 nm ZnO-NP dispersion for 5 h. Later, the membrane was sonicated for 5 min to remove loosely bound particles, resulting in the Janus membrane.

Building on this concept, our study aims to develop a novel Janus membrane through a simple dopamine surface functionalization method on a PP fibrous substrate (Scheme 1). This membrane is designed to serve as an effective barrier against oral bacterial infiltration, incorporating antibacterial properties while facilitating inflammatory fluid decompression. Additionally, it is engineered to possess sufficient tensile strength to withstand oral occlusion conditions, ensuring its practicality in clinical applications.

## 2. Materials and Methods

### 2.1. Materials

Zinc oxide nanoparticles (ZnO-NPs) with average diameters of 30 nm, 50 nm, and 90 nm, as well as dopamine hydrochloride, were purchased from Aladdin (Guangzhou, China). The polypropylene (PP) fibrous substrate, fabricated using the melt-blowing method, was generously provided by a collaborative group at Beijing University of Science and Technology. All chemicals were used as received.

### 2.2. Antibacterial Effect of ZnO-NPs

The antibacterial activity of ZnO-NPs was evaluated using *Streptococcus mutans* ATCC 25175. First, lyophilized bacterial powder was reconstituted in sterile physiological saline, and the bacterial suspension was evenly spread on the surface of sterile brain heart infusion (BHI) agar using a sterile inoculation loop. The plates were then incubated at 37 °C in an anaerobic chamber with an anaerobic gas pack for 24 h. Single colonies were subsequently selected and inoculated into BHI liquid medium, and passaged twice to ensure stable bacterial activity. The final bacterial suspension was adjusted to a concentration of 1 × 10^6^ CFU/mL using a sterile PBS buffer for further experiments.

To assess the antibacterial effect of ZnO-NPs, dispersions of ZnO-NPs with average diameters of 30 nm, 50 nm, and 90 nm were prepared, respectively. ZnO-NPs of different sizes were dispersed in sterile deionized water under ultrasonic conditions to prepare dispersions with concentrations of 5 mg/mL, 2.5 mg/mL, 1.25 mg/mL, and 0.625 mg/mL. These ZnO-NP dispersions were then mixed with *Streptococcus mutans* (*S. m*) suspension (1 × 10^6^ CFU/mL) at a 1:1 volume ratio and cocultured under anaerobic conditions at 37 °C for 24 h. After the incubation, 100 μL of the mixture was spread evenly on the surface of BHI agar in a 90 mm diameter petri dish, followed by an additional 24 h of anaerobic incubation. Colonies were counted and pictures were taken.

### 2.3. Preparation of the PP/Dopamine@ZnO-NP Janus Membrane

Dopamine self-assembles through oxidation to form reactive quinone intermediates that link together to create a polydopamine layer. This process occurs in aqueous solutions, is pH-dependent and allows for strong adhesion to various surfaces [22]. To prepare the dopamine solution, dopamine hydrochloride was dissolved in a 10 mM Tris-HCl buffer (pH 8.5) at a concentration of 2 mg/mL and continuously stirred at room temperature to ensure complete dissolution. The hydrophobic PP fibrous membrane was then floated on the dopamine solution and kept at room temperature for 24 h to allow dopamine to undergo a self-polymerization and form a PDA coating on the membrane surface. After the reaction, the fibrous membrane surface was gently washed with deionized water to remove any unreacted dopamine monomer and byproducts. The PDA-coated fibrous membrane was then immersed in a pre-prepared dispersion of ZnO-NPs with an average diameter of 30 nm. To ensure uniform nanoparticle deposition, the membrane was placed on a horizontal shaker and oscillated at 100 rpm for 5 h. Following the deposition process, the membrane underwent low-frequency ultrasonic treatment (frequency 42 kHz, power 35 W) for 5 min to remove loosely bound particles. Finally, the membrane was vacuum-dried at 60 °C for 12 h to obtain the 30 nm ZnO-modified PP membrane. Therefore, the resulting Janus membrane featured a hydrophobic PP surface on one side and a hydrophilic PP/dopamine@30 nm ZnO-NP surface on the other side, referred to as the PP/dopamine@30 nm ZnO membrane.

### 2.4. Characterization of the Janus Membrane PP/Dopamine@30 nm ZnO

Scanning electron microscopy (SEM) imaging was performed to examine the surface morphology and pore structure of both PP and PP/dopamine@30 nmZnO membranes, enabling the assessment of their microstructural features and surface properties. The microstructure and surface morphology of the fibrous membrane samples were analyzed using a scanning electron microscope (GeminiSEM 300, ZEISS, Oberkochen, Germany). First, the membrane samples from each group were cut into 5 mm × 5 mm pieces and directly adhered to conductive tape. The samples were then gold-coated for 45 s using a Quorum SC7620 sputter coater (Quorum Technologies, Lewes, UK) and observed under vacuum conditions. The surface morphology and pore structure of the PP and PP/dopamine@30 nm ZnO membranes were observed using SEM at an accelerating voltage of 3 kV to assess their microstructural features and surface properties. To evaluate the distribution of ZnO nanoparticles on the membrane surface, elemental composition analysis was conducted using energy-dispersive X-ray spectroscopy (EDX) (XPLORE30, Oxford Instruments, Abingdon, UK). Under an accelerating voltage of 15 kV, elemental mapping was performed to visualize the distribution of oxygen, carbon, and zinc, providing insight into the spatial arrangement of ZnO-NPs on the PP/dopamine@30 nm ZnO membrane surface.

The mechanical properties of the membranes were assessed using a universal testing machine (MTS system, Eden Prairie, MN, USA). Rectangular samples (*n* = 3) of each membrane were prepared and positioned between two grips for tensile testing at a constant crosshead speed of 10 mm/min at room conditions. The Young’s modulus was determined from the initial linear region (0–10%) of the stress–strain curve.

The hydrophilicity/hydrophobicity of the membranes was evaluated by measuring their dynamic contact angles using the inclined plate method with a surface tension measurement instrument (SZ-CAMC32, Shanghai Xuanzhun, Shanghai, China). Unmodified and ZnO-modified fibrous membrane samples were cut into 1 cm × 1 cm pieces and fixed onto the inclined plate device. A 5 μL of deionized water was droplet-deposited onto the membrane surface using a micropipette, and the dynamic behavior of the droplets was recorded in real time using a CCD camera. The advancing contact angle (ACA) and receding contact angle (RCA) were determined, and hysteresis between the two angles was calculated. Each sample was measured three times at different positions, and the average value was taken as the final result to ensure data reliability and reproducibility.

The water flux of the membrane was tested using a dead-end filtration device at varying pressures. Prior to testing, the Janus membrane was wetted with anhydrous ethanol and then pre-pressurized at 0.1 MPa for 30 min. Water flux measurements were performed at pressures of 0.01 MPa, 0.02 MPa, 0.03 MPa, 0.04 MPa, and 0.05 MPa. During the test, the amount of distilled water that permeated through the membrane from the PP/dopamine@30 nm ZnO side to the PP side was recorded along with the collection time. Triplicate measurements were taken at different positions for each sample, and the average value was used as the final result ensure measurement accuracy and reproducibility.

### 2.5. Cytotoxicity Test In Vitro

Third molars and premolars without signs of periodontal or carious disease were extracted from healthy individuals aged 18–25 years at Beijing Stomatological Hospital, Capital Medical University, with informed consent obtained from all donors. Human dental pulp stem cells (hDPSCs) were isolated and cultured according to a previously published method [23]. PP membrane and PP/dopamine@30 nm ZnO membranes were cut into 1 cm × 1 cm squares, sterilized, and fixed into CellCrown^TM^ inserts (Sigma-Aldrich, St. Louis, MO, USA) before use. To assess the cytotoxicity of the membranes, hDPSCs were seeded into 24-well plates at a density of 5 × 10^4^ cells/mL. The membrane-containing CellCrown^TM^ inserts were placed into the wells and immersed in complete medium for 1 and 3 days. Cell viability was then evaluated using the Cell Counting Kit-8 assay (Dojindo, Kumamoto, Japan), with absorbance measured at 450 nm using a microplate reader. A positive control group containing a CellCrown^TM^ insert without a membrane was included for comparison. Relative growth rate percentage (RGR%) was calculated as follows:

$$\mathrm{RGR}\;(\%)=\frac{OD-ODblank}{ODpositivecontrol-ODblank}\times 100\%$$

### 2.6. In Vitro Antibiofilm Performance

*Streptococcus mutans* ATCC 25175 was used as the model strain to assess the antibacterial performance of the fibrous membranes. A bacterial suspension (2 × 10^3^ CFU/mL) was evenly inoculated onto the surface of the PP and PP/dopamine@30 nmZnO membranes and incubated at 37 °C for 3 days. Following incubation, the membrane samples were divided into two groups. One group was immersed in a 2.5% glutaraldehyde solution at 4 °C for 24 h to fix the bacterial cells, followed by dehydration through a graded ethanol series (30%, 50%, 70%, 90%, and 100%), with each concentration for 10 min. The dehydrated samples were gold-coated and observed under a SEM to examine the morphology and structure of the bacterial biofilm. The SEM images were analyzed using ImageJ software (version 1.54g) to quantitatively assess the coverage area of the bacteria on the membrane surface. The other group was subjected to live/dead staining with LIVE/DEAD Baclight bacterial viability kit (ThermoFisher, Waltham, MA, USA). Samples were stained with SYTO9 (green fluorescence for all bacteria) and propidium iodide (PI, red fluorescence for membrane-damaged bacteria) for 30 min in the dark. The stained samples were then observed under a fluorescence microscope (Nikon, Tokyo, Japan) to assess bacterial viability.

### 2.7. In Vivo Bacterial Barrier Ability

All animal experiments were conducted following approval from the Ethics Committee on Animal Welfare of Capital Medical University (Ethics No. AEEI-2023-265). The pulp chamber defect model was established based on our previous protocol [24]. Twenty-month-old female miniature pigs were used for the experiment. General anesthesia was administered via intravenous injection through the ear vein. A high-speed turbine handpiece with an EX31 bur was used to create a 3–4 mm pulp-exposing hole at the center of the occlusal surface. A gelatin sponge (Xiang’en, Nanchang, China) was placed beneath the defect, and an unmodified PP fibrous membrane and Janus-modified PP/dopamine@30 nm ZnO fibrous membrane were placed over the opening. The membranes were sealed with flowable resin (Dentsply Sirona, Charlotte, NC, USA). After 3 days, the membranes were carefully removed, fixed in 2.5% glutaraldehyde for 24 h at 4 °C, dehydrated in a gradient ethanol series, and gold-coated. SEM was used to examine surface morphology and bacterial infiltration.

### 2.8. Statistical Analysis

All experimental data were statistically analyzed using GraphPad Prism 9 software. Each experimental group was performed with at least three independent replicates, and results are presented as mean ± standard deviation (mean ± SD). Before statistical analysis, the normality of the data was assessed using the Shapiro–Wilk test, and homogeneity of variance was verified using the Levene’s test. For normally distributed data with homogenous variances, an independent samples t-test was used to compare differences between the two groups. For data that did not follow a normal distribution, the Mann–Whitney U test was applied. Statistical significance was set at *p* < 0.05, with significance levels indicated as follows: * *p <* 0.05, ** *p <* 0.01, *** *p <* 0.001, **** *p <* 0.0001.

## 3. Results and Discussion

### 3.1. Evaluation of the Antibacterial Effect of ZnO-NPs with Varying Diameters

**Figure 1:**
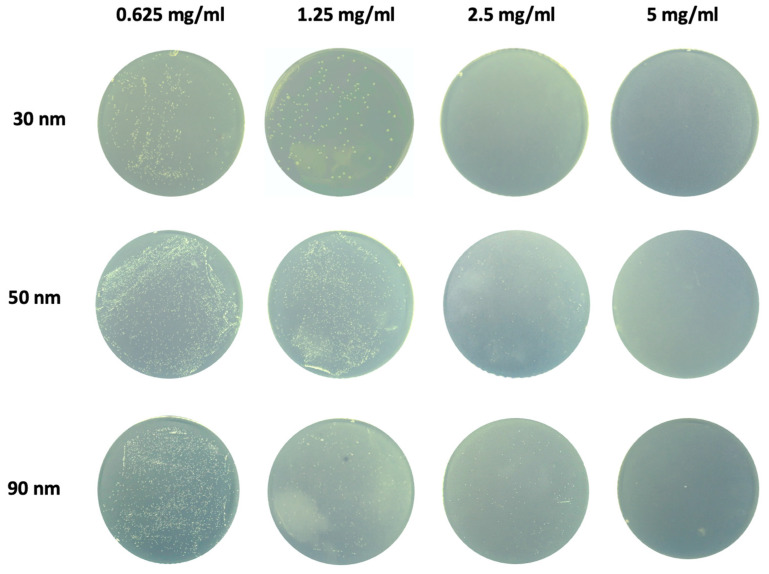
Screening of antibacterial effect of ZnO-NPs with different particle sizes. The antibacterial activity of ZnO-NPs with varying diameters (30 nm, 50 nm, and 90 nm) against *S. mutans* was evaluated using the colony counting method.

As aforementioned, antibacterial property is critical in decompressing inflammatory fluid. The antibacterial activity of ZnO-NPs with different particle sizes (30 nm, 50 nm, and 90 nm) against *S. mutans* was evaluated using the colony counting method. ZnO-NPs with a diameter of 30 nm exhibited the strongest antibacterial effect at the same concentration (Figure 1), resulting in significantly lower CFU scores than those in the 50 nm and 90 nm groups. This enhanced antibacterial performance may be attributed to the larger specific surface area of the 30 nm nanoparticles, which increases bacteria contact and surface energy, leading to the generation of more reactive oxygen species (ROS) [25]. The higher ROS production enhances antibacterial activity by causing oxidative stress in bacterial cells [26]. In addition, smaller nanoparticles are more likely to penetrate the bacterial cell wall and enter the bacteria, thereby inhibiting metabolism or damaging bacterial organelles. Based on these findings, 30 nm ZnO-NPs were selected for surface modification of the PP/dopamine@ZnO membrane in subsequent experiments.

### 3.2. Fabrication and Characterization of the PP/Dopamine@ZnO Membrane

**Figure 2:**
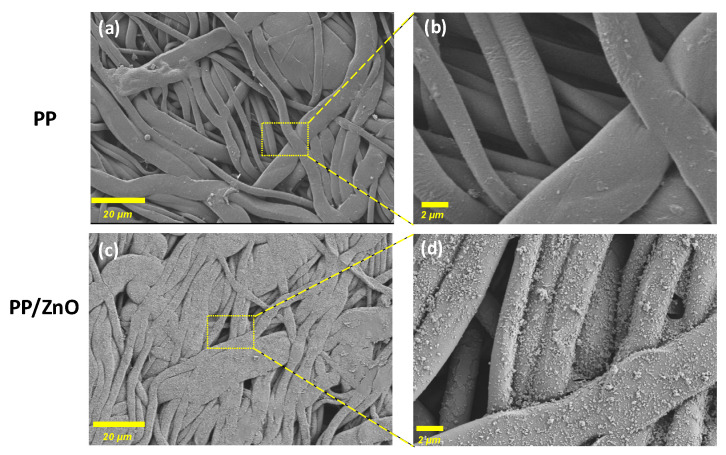
SEM images of PP and PP/dopamine@30 nm ZnO membrane: (**a**,**c**) ×500, (**b**,**d**) ×5000, respectively.

SEM analysis of the unmodified PP fibrous membrane revealed a relatively regular fiber structure with noticeable variations in fiber diameter and visible pore distribution (Figure 2a,b). After ZnO modification, the PP/dopamine@ZnO surface displayed distinct nanoscale protrusions, indicating successful nanoparticle deposition (Figure 2c,d).

**Figure 3:**
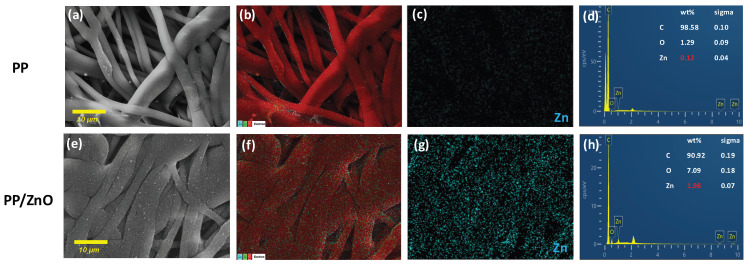
EDX mapping and elemental analysis of PP and PP/dopamine@30 nmZnO membrane: (**a**,**e**) ×1000, SEM images of PP and PP/dopamine@30 nm ZnO; (**b**,**f**) ×1000, EDX elemental merging images; (**c**,**g**) the zinc element distribution; (**d**,**h**) EDX spectra of the PP and PP/dopamine@30 nm ZnO membrane.

To ensure the stable attachment of ZnO-NPs and eliminate loosely bound particles, low-frequency ultrasonic cleaning was performed as the final preparation step. To verify ZnO retention on the membrane post-treatment, EDX analysis was conducted. The results confirmed a uniform distribution of Zn across the fiber bundles (Figure 3g), with a measured Zn weight percentage of 1.98% (Figure 3h). The strong Zn signal in the EDX spectrum indicates stable ZnO-NP binding, reinforcing the antibacterial properties of the membrane material. The pure PP membrane exhibited a 0.12% weight percentage of Zn, which is attributed to a false positive signal (Figure 3a–d).

### 3.3. Tensile Stress of PP/Dopamine@ZnO Membranes

**Figure 4:**
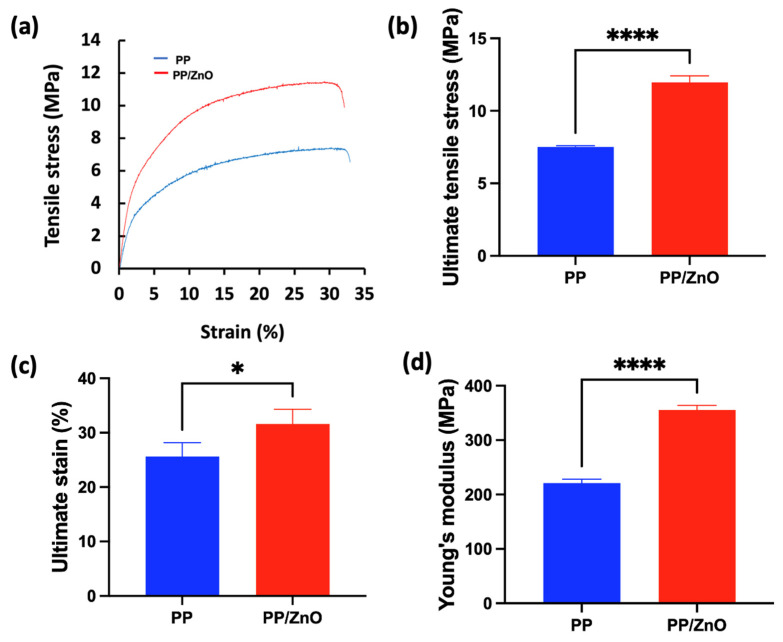
Tensile stress–strain curves and analysis of PP and PP/dopamine@ZnO membrane: (**a**) stress–strain curves of PP and PP/dopamine@30 nm ZnO membranes; (**b**–**d**) the mechanical properties obtained from the tensile test including (**b**) ultimate tensile stress, (**c**) ultimate strain, and (**d**) Young’s modulus. * *p* < 0.05, **** *p* < 0.0001.

Tensile testing revealed that the incorporation of ZnO significantly enhanced the mechanical properties of the PP membrane. (Figure 4). The Young’s modulus increased from 211.1 MPa for the pure PP membrane to 355.5 MPa for the ZnO-coated membrane (*p* < 0.0001), indicating improved stiffness. Additionally, the ultimate tensile strength rose from 7.5 MPa to 12.0 MPa (*p* < 0.0001), and the elongation at break also showed a substantial increase, reflecting enhanced ductility and strength. The effect of ZnO coatings on the mechanical properties of substrates, however, varies significantly depending on the coating preparation method and nanoparticle characteristics [27,28]. When ZnO-NPs are directly immersed or used in combination with a binder, the strength and elastic modulus of the ZnO films tend to decrease. In contrast, the tensile strength and elongation at break of ZnO-coated membranes are significantly improved when a wax emulsion is incorporated into the coating formulation [29].

### 3.4. Fluid Management Ability of PP/Dopamine@ZnO Membrane

**Figure 5:**
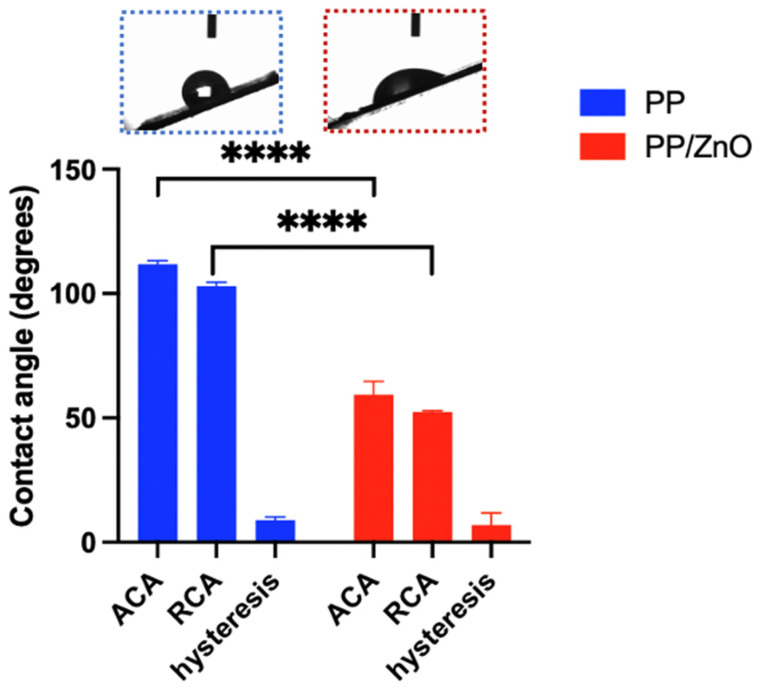
Advancing contact angle (ACA), receding contact angle (RCA) and hysteresis of PP and PP/dopamine@30 nm ZnO membrane samples. **** *p* < 0.0001.

The contact angle is a key indicator of surface wettability, with lower angles signifying stronger hydrophilicity, which improves liquid interaction and filtration performance. The unmodified PP membrane was hydrophobic, evidenced by an ACA of 111.86° ± 1.10° and RCA of 102.99° ± 1.25° (Figure 5). Following ZnO modification, the PP/dopamine@ZnO membrane became hydrophilic, achieving an ACA of 59.36° ± 4.39° and an RCA of 52.42° ± 0.42°, respectively. This shift from hydrophobic to hydrophilic suggests that ZnO modification significantly enhances the membrane’s wettability. The increase in hydrophilicity can be attributed to the surface properties of ZnO and its interaction with dopamine, which collectively increase the hydrophilicity of the membrane. Enhanced hydrophilicity plays a vital role in improving exudate filtration. A more hydrophilic surface allows liquids to better contact and permeate through the fibrous membrane, thereby enhancing filtration efficiency. Therefore, the reduction in water contact angle further confirms the significant improvement in fibrous membrane performance after ZnO modification.

### 3.5. Water Flux Experiment of PP/Dopamine@30 nm ZnO Membrane

**Figure 6:**
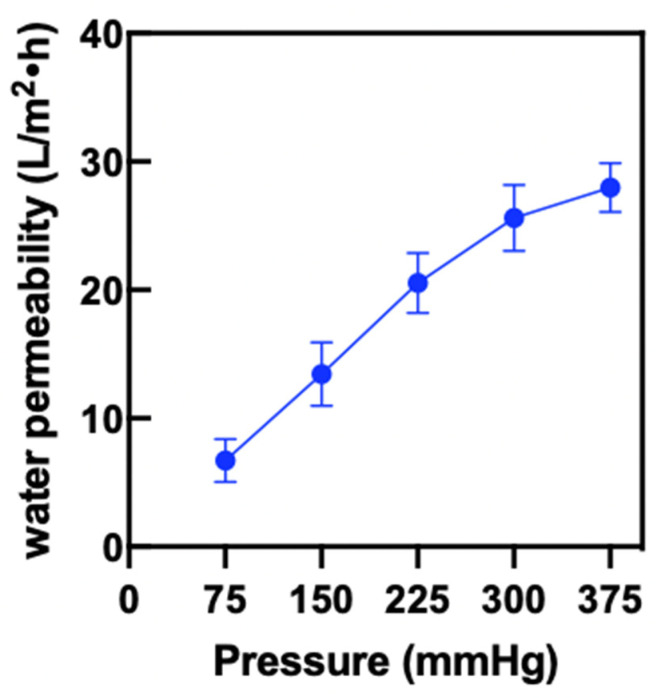
The pure water flux on PP/dopamine@30 nm ZnO membrane.

Permeation flux, defined as the volume of liquid passing through a unit area per unit time, is a key parameter for evaluating the filtration performance of a material as it reflects the rate at which the material allows liquid to pass through. As shown in Figure 6, the permeation flux of the Janus membrane exhibits a nearly linear relationship with pressure in the range of 75 mmHg to 375 mmHg. This indicates that the surface of the Janus membrane is relatively uniform, and the pore structure and permeation process are stable. At a pressure of 75 mmHg, the water flux is 6.72 ± 1.36 L/(m^2^·h), while at 375 mmHg, the water flux increases to 27.99 ± 1.56 L/(m^2^·h). These results confirm the reliable and efficient filtration performance of the Janus membrane across varying pressure levels.

### 3.6. Biocompatibility of PP/Dopamine@30 nm ZnO Membrane

**Figure 7:**
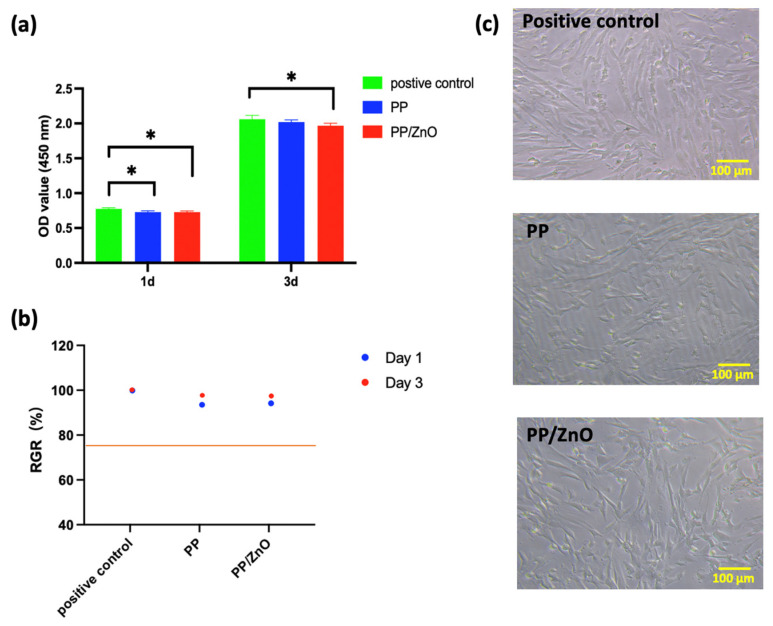
Biocompatibility of PP/dopamine@30 nm ZnO membrane: (**a**) cell viability of hDPSCs direct contact with PP and PP/ddopamine@30 nm ZnO membranes; (**b**) relative growth rate percentage of (**a**); (**c**) bright-field microscopy images of hDPSCs in direct contact without and with different membrane surfaces after 24 h of culture. * *p* < 0.05.

After coculturing hDPSCs with the membranes for 1 and 3 days, the PP membrane on day 1 and the PP/dopamine@30 nm ZnO membranes on both day 1 and day 3 exhibited significantly lower cell viability compared to the control (*p* < 0.05) (Figure 7). However, the relative growth rates (RGR%) in all groups remained above 75%, indicating that the membranes met the cytocompatibility criteria for biomedical applications, as defined by ISO 10993 [30].

### 3.7. Antibacterial Ability of PP/Dopamine@ZnO Membrane In Vitro

**Figure 8:**
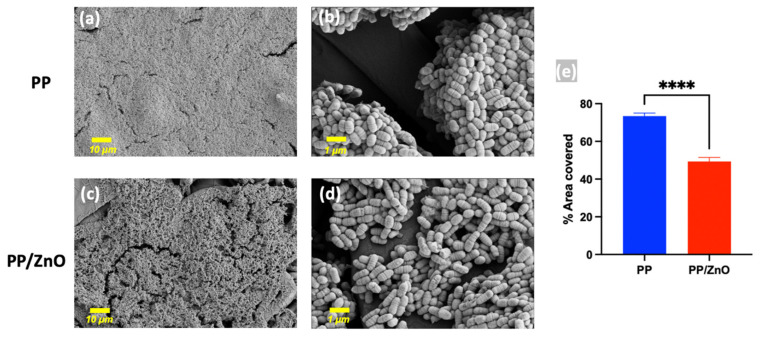
SEM images of *S. mutans* on membranes. The anti-biofilm effect against *S. mutans* on the surfaces of PP (**a**,**b**) and PP/dopamine@30 nm ZnO (**c**,**d**) membrane; (**e**) quantitative measurement of area covered by biofilm on SEM. **** *p* < 0.0001.

**Figure 9:**
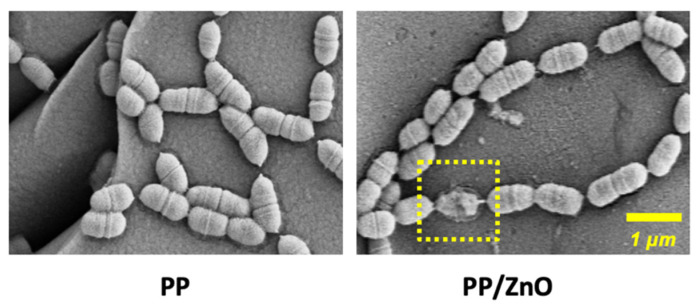
SEM images of *S. mutans* on PP and PP/dopamine@30 nmZnO surfaces. The yellow dashed box indicates the disruption of the *S. mutans* membrane.

**Figure 10:**
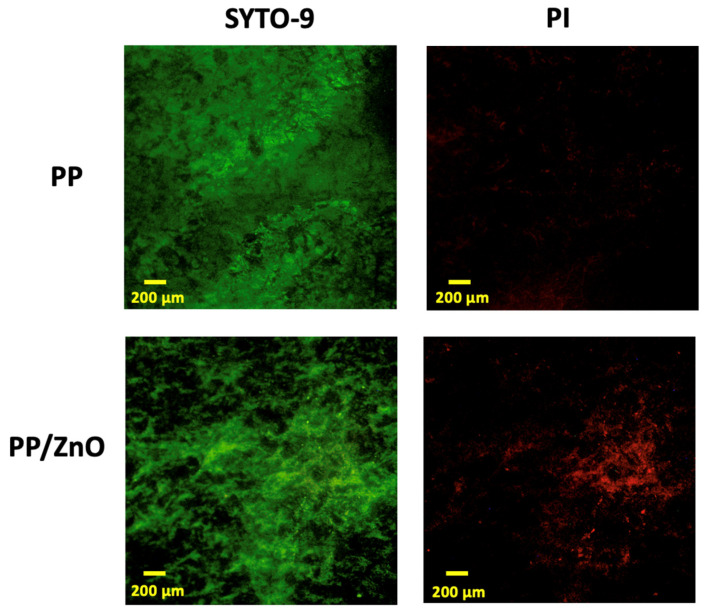
Biofilm inhibition assessments. Live/dead BacLight staining of biofilms of *Streptococcus mutans* cultured on PP and PP/dopamine@30 nm ZnO membranes for 24 h.

To evaluate the antibacterial performance, we conducted in vitro experiments by coculturing *S. mutans* with PP and PP/dopamine@ZnO membranes and observing biofilm formation. The results showed that a thick and dense bacterial biofilm formed on the surface of the PP membrane, with bacteria accumulating in large quantities and pores being clogged (Figure 8a,b). In contrast, on the PP/dopamine@ZnO membrane, the thickness of the bacterial biofilm was significantly reduced, and the pores remained unobstructed by the biofilm (Figure 8c,d). SEM analysis showed that the bacterial coverage rate decreased from 73.46% ± 1.58% on the PP membrane to 49.34% ± 2.15% on the PP/dopamine@ZnO membrane (*p* < 0.0001) (Figure 8e). This indicates that the ZnO-modified PP membrane exhibited significant antibacterial advantages, effectively inhibiting bacterial growth while maintaining the pore structure of the membrane. This feature is especially beneficial where high internal pressure requires liquid to permeate outward, as it results in lower filtration resistance, facilitating liquid release. Additionally, the reduced ability of bacteria to form biofilms also indirectly extends the filtration time and improves the membrane’s service life. The antibacterial effect of ZnO may be attributed to the local contact of ZnO with the bacterial cells, leading to the internalization of the cell wall, increased membrane permeability, and the uptake of harmful dissolved zinc ions [31]. As shown in Figure 9, SEM images reveal that the integrity of the *S. mutans* cell wall was disrupted on the PP/dopamine@ZnO membrane, resulting in bacterial death. To further assess the antibacterial effect of the PP/dopamine@30 nm ZnO membrane, a live/dead staining assay was performed. SYTO9, a membrane-permeable dye, stained the total bacterial population, while propidium iodide (PI), which only penetrates damaged membranes, selectively labeled non-viable cells. After 24 h of coculture with the PP/dopamine@30 nm ZnO membrane, *S. mutans* were co-stained with SYTO9 and PI. The results showed strong red fluorescence in the PP/dopamine@30 nm ZnO group, indicating substantial membrane damage to *S. mutans* (Figure 10).

### 3.8. Barrier Ability of PP/Dopamine@30 nm ZnO In Vivo

**Figure 11:**
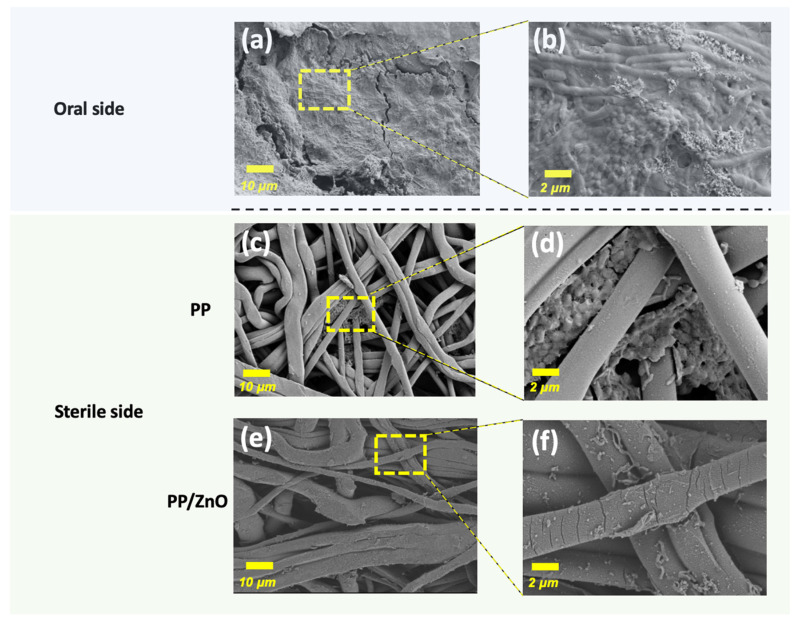
SEM images exhibiting the anti-penetration effect of membranes placed on minipig premolars for 3 days: (**a**,**b**) oral side of the membrane; (**c**,**d**) sterile side of PP membrane after 3 days bacteria penetration; (**e**,**f**) sterile side of Janus membrane (PP/dopamine@30 nm ZnO surface) after 3 days bacteria penetration (Note: the oral side of both the PP and Janus membranes is identical, thus only one group of images is provided).

The oral microbiome is highly complex, and when membrane materials are exposed to the oral cavity, bacteria can migrate from the oral side to the sterile side, potentially causing infection, inflammation, and impaired tissue healing. Therefore, evaluating the ability of the PP/dopamine@30 nm ZnO membrane to act as a bacterial barrier is essential. In a 3-day study with miniature pigs, where the membranes were applied to the occlusal surface of the premolars, SEM analysis was conducted to evaluate bacterial presence. On the oral-exposed side (Figure 11a,b), large amounts of bacteria colonization and biofilm formation were observed on the fibrous membrane, including cocci and bacilli. On the unexposed sterile side, bacteria migrated from the fibrous intersections on the PP membrane in a layered arrangement (Figure 11c,d), while on the PP/dopamine@30 nm ZnO membrane, only a few distorted and incomplete bacteria were adhered to the surface (Figure 11e,f). These findings suggest that, although the PP/dopamine@30 nm ZnO membrane has antibacterial properties, it does not fully prevent bacterial migration within 3 days. Therefore, for applications requiring strict sterility, such as vital pulp preservation in irreversible pulpitis, the exposure time of the PP/dopamine@30 nm ZnO membrane in the oral cavity should be limited to less than 3 days. The issue of bacterial invasion through fibrous membrane warrants series attention. Previous studies have also investigated the barrier function of such membranes and confirmed that, when exposed in oral condition, microorganisms could penetrate the membrane and negatively impact the healing process [32,33]. Consequently, further improvement of the barrier properties of fibrous membranes is necessary to ensure better clinical outcomes.

In previous studies using ZnO nanoparticles for guided bone regeneration or guided tissue regeneration applications, ZnO was utilized for its dual antibacterial and osteogenic properties. ZnO has been demonstrated to enhance osteoblast differentiation and exhibit significant antibacterial effects against both planktonic bacteria and established biofilms, thereby helping to prevent infection [34,35]. Additionally, some studies have highlighted that the porous membranes and ZnO with increased hydrophilicity, can enhance tissue integration, facilitate blood infiltration, and stabilize blood clots, contributing to tissue repair [36]. However, limited research has focused on the drainage capacity of such membranes. In contrast, our PP/ZnO membrane is engineered for use in oral applications such as pulpitis, where efficient exudate filtration and robust antibacterial protection are critical. Our design features a Janus structure with a hydrophilic side that filters exudates from pulpitis and a hydrophobic side that prevents bacterial adhesion, effectively blocking bacterial penetration. This dual-functionalized membrane integrates both antibacterial properties and exudate filtration in a single material, with the added function of bacterial exclusion, offering it a novel and effective solution for oral applications.

Dopamine undergoes self-polymerization under mildly alkaline conditions (typically around pH 8.5), during which it is oxidized to form reactive quinone intermediates. These intermediates then undergo intramolecular and intermolecular reactions, leading to the formation of a cross-linked PDA coating [22]. However, PDA coatings are known to be susceptible to enzymatic degradation, particularly by enzymes such as laccase, raising concerns about their long-term stability in vivo [37]. This inherent degradability may affect the sustained functionality of dopamine-based surface modifications in biological environments. In our study, cytotoxicity was assessed by direct contact between the membranes and hDPSCs using a CCK-8 assay. Both PP and PP/dopamine@30 nm ZnO membranes exhibited no cytotoxic effects after 1 and 3 days of incubation, suggesting that any potential degradation products, including polydopamine fragments or ZnO nanoparticles, were not harmful under these in vitro conditions. Although our results demonstrate good initial biocompatibility, the long-term stability and degradation behavior of dopamine coatings in vivo warrant further investigation to ensure their reliability in clinical applications.

## 4. Conclusions

In this study, we successfully developed a Janus membrane with antibacterial and filtration properties. Using a straightforward floating immersion method, a dopamine@30 nm ZnO coating was formed on the PP substrate, resulting in an asymmetric bilayer structure with hydrophobic PP on one side and hydrophilic PP/dopamine@30 nm ZnO on the other. This design enabled liquid filtration through the barrier membrane while preventing bacterial invasion and liquid penetration from the oral side. The addition of ZnO significantly improved the mechanical properties of the PP membrane, enhancing its stiffness, tensile strength, and ductility. Furthermore, the incorporation of ZnO on the PP membrane endowed it with antibacterial properties, significantly inhibiting the formation of bacterial biofilms by *S. mutans* in vitro. In miniature pig animal experiments, the sterile side of the PP/dopamine@30 nm ZnO membrane showed fewer bacteria. These findings indicate that this material holds great potential for applications in oral exudate filtration, such as in cases of irreversible pulpitis, effectively extending filtration time and reducing the risk of oral infections.

## Data Availability

Data are contained within the article.
